# Optimum Conditions for the Fabrication of Zein/Ag Composite Nanoparticles from Ethanol/H_2_O Co-Solvents Using Electrospinning

**DOI:** 10.3390/nano6120230

**Published:** 2016-12-01

**Authors:** Seong Baek Yang, Mohammad Mahbub Rabbani, Byung Chul Ji, Dong-Wook Han, Joon Seok Lee, Jong Won Kim, Jeong Hyun Yeum

**Affiliations:** 1 Department of Advanced Organic Materials Science and Engineering, Kyungpook National University, Daegu 41566, Korea; ysb@knu.ac.kr (S.B.Y.); liangst@naver.com (M.M.R.); bcji@knu.ac.kr (B.C.J.); 2 Department of Cogno-Mechatronics Engineering, Pusan National University, Busan 46241, Korea; nanohan@pusan.ac.kr; 3 Department of Textile Engineering & Technology, Yeungnam University, Gyeongsan 38541, Korea; leejs@ynu.ac.kr

**Keywords:** zein, silver, nanoparticles, electrospinning, antibacterial activity

## Abstract

The optimum conditions for the fabrication of zein/Ag composite nanoparticles from ethanol/H_2_O cosolvents using electrospinning and the properties of the composite were investigated. The zein/Ag nanoparticles were characterized using field-emission scanning electron microscopy, transmission electron microscopy (TEM), X-ray diffraction (XRD), and thermogravimetric analysis. The antibacterial activity of the zein/Ag composite nanoparticles was also investigated. The XRD patterns and TEM images indicate the coexistence of a zein matrix and well-distributed Ag nanoparticles.

## 1. Introduction

Polymer nanocomposites can exhibit beneficial properties that cannot be attained from their individual components. Inorganic nanomaterials are generally dispersed in an organic polymer matrix to prepare these nanocomposites by simply mixing the required organic and inorganic components. The introduction of inorganic nanoparticles into the polymer matrix has been demonstrated to be an effective and low-cost method of improving the performance of polymer materials [1,2,3,4,5,6].

Zein is the major storage protein in corn. It is hydrophobic, renewable, biodegradable, exhibits low toxicity, and is a relatively straightforward biopolymer that can be prepared using electrospinning [7,8,9,10,11,12]. Alcohols are excellent solvents for zein; however, a water-ethanol mixture has been shown to be the best solvent for zein [13,14,15,16,17,18]. The major structure of zein is a helical wheel conformation in which nine homologous repeating units are arranged in an anti-parallel form stabilized by hydrogen bonds. The presence of two important polar amino acids, proline and glutamine, is responsible for the good cell compatibility and additional hydrophobic characteristics of zein compared to other proteins. However, the high proportion of non-polar amino acid residues in zein controls its solubility [8,9,19]. Since zein can form tough, glossy, hydrophobic coatings and exhibits antibacterial activity, it is used in the food industry. Zein is also used in the manufacture of plastics, paper coatings, textiles, adhesives, substitutes for shellac, laminated board, and solid color printing films. In the pharmaceutical industry, zein is widely used for coating capsules for protection, controlled-release drugs, and masking of flavors and aromas [9,18].

Ag nanoparticles are widely used as photosensitive components and catalysts. They are used to fight infections and prevent spoilage. Due to their comparatively high safety, as well as antibacterial and multi-functional properties, many researchers have successfully developed antibacterial and disinfectant agents that combine Ag nanoparticles with various polymers. Most microorganisms are not resistant to Ag nanoparticles. Ag nanoparticles have an extremely large specific surface area and can significantly increase the antibacterial efficiency [5,20,21,22,23,24].

Electrospinning is a unique and convenient method of producing nanofibers from various polymers for a wide range of applications [11,25,26,27,28,29,30,31,32,33]. Other nanostructures, such as nanoparticles and nanobeads, can be obtained using electrospinning depending on the processing conditions [10]. These nanometer-scale electrospun materials exhibit excellent properties that cannot be attained from their bulk counterparts [31,32]. In the electrospinning process, a high voltage is applied to a polymer solution to create electrically-charged jets. The structures and properties of the electrospun end products strongly depend on certain variables related to the polymer solution and/or processing parameters. Nanostructured materials with different morphologies can be produced by controlling these variables [27,31,32,34]. There are many parameters that will influence the morphology of the resultant electrospun fibers, which range from beaded fibers to fibers with pores on their surface. The parameters affecting electrospinning and the fiber mat are broadly classified into polymer solution parameters and processing conditions that include the applied voltage, temperature and effect of the collector, and ambient conditions. With an understanding of these parameters, it is possible to create nanofibers with different morphologies by varying these parameters. Electrospinning is a complicated process that involves solvent diffusion/evaporation/cooling, heat transfer, water condensation, and polymer diffusion, in addition to the operation variables [35,36]. Khanum et al. reported on the morphology control of a thiophene derivative through electrospinning using various solvents [37]. In the same study, they investigated the influence of various solvents on the crystallite size of thiophene derivatives using electrospinning and suggested their potential as drug carrier hollow microspheres and for obtaining a spiked morphology. Our group investigated the effect of polymer concentration, tip-to-collector distance, and applied voltage in an electrospinning system. We prepared poly(vinyl alcohol) microfibers, poly(vinyl alcohol)/chitosan oligosaccharide/clay nanofibers, pullulan/poly(vinyl alcohol)/montmorillonite nanofibers, etc., by controlling the electrospinning conditions and characterized them [38,39,40].

Since Ag nanoparticles are widely used in various polymer composites to improve their physicochemical properties, and hence expand their applications, the aim of this study was to evaluate the effects of Ag content on the morphologies and properties of the zein/Ag composite nanoparticles prepared from aqueous solutions using electrospinning. The effect of the Ag nanoparticles on the thermal properties and morphologies of the zein nanoparticles was examined using thermogravimetric analysis (TGA), field-emission scanning electron microscopy (FE-SEM), transmission electron microscopy (TEM), and X-ray diffraction (XRD). Furthermore, this study demonstrates the effectiveness of the zein/Ag composite nanoparticles in terms of antibacterial performance, which provides an opportunity for developing a new preservative and may have medical applications.

## 2. Experimental

### 2.1. Materials

All of the chemicals used in this study were used as received without further purification. Zein obtained from corn (molecular weight = 35,000) was purchased from the Tokyo Chemical Industry Co. Ltd., Tokyo, Japan, and 96% (*v/v*) ethanol was obtained from Daejung Chemical and Materials Co. Ltd., Daejeon, Korea. An aqueous Ag nanoparticle dispersion (AGS-WP001 10,000 ppm) with particle diameters of approximately 15–30 nm was obtained from Miji Tech., Ansan, Korea. Doubly-distilled water was used with ethanol as a co-solvent to prepare all of the solutions.

### 2.2. Preparation of Zein/Ag Blend Solutions

The zein/Ag blend solutions for electrospinning were prepared as reported previously [41]. To optimize the pure zein nanomaterials, the zein solutions were prepared by dissolving zein at different volume ratios of ethanol/water, and various zein concentrations were used (10, 15, 20 wt %). Afterward, variable amounts (2 and 4 wt % based on the final solution concentration) of Ag nanoparticle dispersions were added separately to the zein solutions at 25 °C with continuous stirring for another 1 h.

### 2.3. Electrospinning Process

Nanoparticles were prepared by electrospinning the pure zein and zein/Ag blend solutions. During electrospinning, a voltage of 15 kV (Chungpa EMT Co. Ltd., Seoul, Korea; model CPS-60K02VIT) was applied to the zein/Ag solution contained in a syringe via an alligator clip attached to the syringe needle. The solution was delivered to a blunt needle tip via a syringe pump to control the solution flow rate. The prepared nanoparticles were collected on an electrically-grounded piece of Al foil placed at a vertical distance of 15 cm from the needle tip. The needle size (length = 20 mm, diameter = 1 mm) was controlled on the spinneret for electrospinning. For electrospinning, the flow rate was 0.04 mL/h and was performed at 25 °C and 60% relative humidity.

### 2.4. Characterization

The morphologies of the electrospun zein and zein/Ag composite nanoparticles were examined using FE-SEM and TEM. Photoshop CS 6 (Adobe, San Jose, CA, USA) was used to measure the average diameter and at least 25 different particles and 100 different segments were randomly selected from each image. The FE-SEM images were collected using a JEOL JSM-6380 microscope (JEOL, Peabody, MA, USA) after coating with Au. The TEM analysis was conducted on a Hitachi H-7600 machine (Hitachi, Tokyo, Japan) with an accelerating voltage of 100 kV. Reflection-type XRD (Philips model X’Pert APD) using Cu Kα radiation with a wavelength of 0.154 nm was used to investigate the crystallinity of the composite nanoparticles. The viscosity of zein and zein/Ag solution was measured using a viscometer (A & D Ltd., Tokyo, Japan, SV-10) at 25 °C. The thermal stability of the zein/Ag nanoparticles was studied using TGA (TA Instruments, New Castle, DE, USA, Q-50) at a heating rate of 10 °C/min from room temperature to 600 °C under a nitrogen gas atmosphere. The antibacterial performance of the zein/Ag composite nanoparticles was investigated against *Staphylococcus aureus* (ATCC6538) using KSM 0146 (the shake flask method). The release of Ag^+^ ion by the zein/Ag nanoparticles was monitored by inductively coupled plasma spectrophotometer (PerkinElmer, Waltham, MA, USA, Optima 7300DV) analyzing solutions obtained by the interaction of the solvent with the nanoaprticles samples at different time.

### 2.5. Anti-Microbial Performance Test

Anti-bacterial performance of zein/Ag nanoparticles was investigated using *Staphylococcus aureus* (ATCC6538). Samples were prepared by dispersing the nanoparticles into a viscous aqueous solution containing 0.05 wt % of polyoxyethylenesorbitan monooleate (Sigma Aldrich, St. Louis, MO, USA tween 80). A mixed culture of microorganisms was obtained on tryptone soya broth after 18 h of incubation at 32 °C. Then, 0.5 g of sample was inoculated with 0.2 g of the microorganism suspension to adjust the initial concentration of bacteria to 10^6^ CFU/g. Subsequently, the inoculants were mixed homogeneously with the samples and stored at 32 °C. The microbial counts were performed using the pour plate count method.

## 3. Results and Discussion

### 3.1. Particle Morphology

The diameter of electrospun nanomaterials plays a key role in their final properties. It is well known that the diameter and morphology of electrospun nanomaterials are strongly dependent on several processing parameters [31] including the polymer conformation, solution viscosity, elasticity, electrical conductivity, polarity, and surface tension of the solvent. Moreover, operational conditions, such as the strength of the applied electric field, distance between the spinneret and the collector, and the feed rate of the polymer solution also affect the characteristics of the electrospun nanomaterials. In addition to these variables, the humidity and temperature of the surroundings may also play an important role in determining the morphology and diameter of the electrospun nanomaterials.

**Figure 1:**
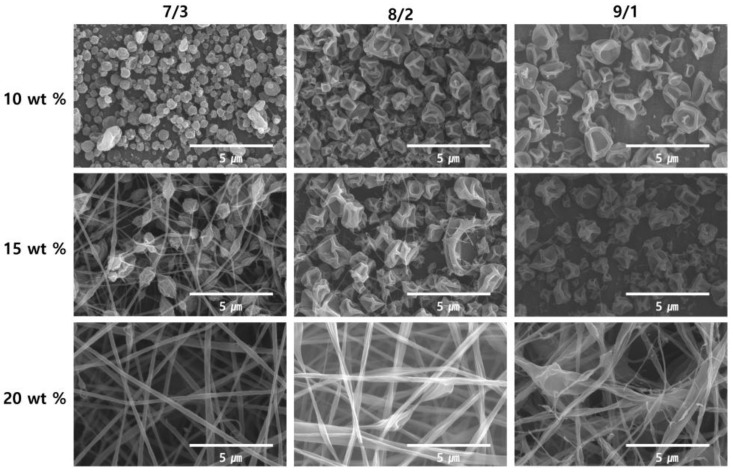
Field emission scanning electron microscopy (FE-SEM) images of zein nanomaterials electrospun from ethanol aqueous solutions with ethanol/water ratios of 7/3, 8/2 and 9/1 (*v/v*) at polymer concentrations of 10, 15 and 20 wt % (Tip-to-collector distance (TCD) = 15 cm and applied voltage = 15 kV).

**Figure 2:**
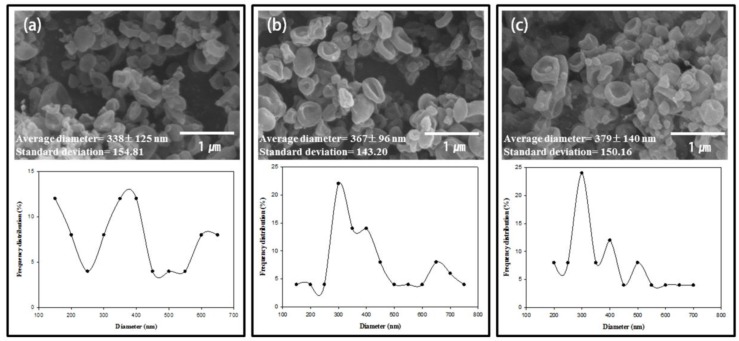
FE-SEM images, average diameter, and distribution of zein/Ag nanoparticles electrospun from ethanol aqueous solutions with an ethanol/water ratio of 8/2 (*v*/*v*) at Ag concentrations of (**a**) 0, (**b**) 2 and (**c**) 4 wt % (polymer concentration = 10 wt %, TCD = 15 cm, and applied voltage = 15 kV).

**Figure 3:**
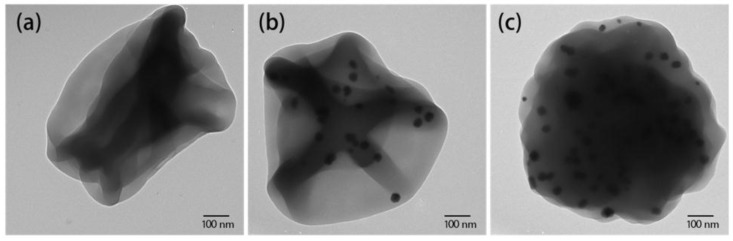
Transmission electron microscopy images of zein/Ag nanoparticles electrospun from ethanol aqueous solutions with an ethanol/water ratio of 8/2 (*v/v*) at Ag concentrations of (**a**) 0, (**b**) 2 and (**c**) 4 wt % (polymer concentration = 10 wt %, TCD = 15 cm, and applied voltage = 15 kV).

Figure 1 shows the variation in the morphologies, imaged using FE-SEM, of the 10, 15 and 20 wt % zein electrospun nanomaterials prepared from the 7/3, 8/2 and 9/1 (*v/v*) ethanol/water co-solvents. At a low zein concentration (10 wt %), beads with uniform shape and size were obtained. As the concentration and viscosity of the zein solutions increased (Table 1), the morphology of the nanomaterials changed. Zein with a higher viscosity tended to attain a highly elongated shape, like a fibrous structure, more easily than zein at low concentration. This suggests that increasing the entanglement of zein chains by increasing the concentration contributes to the formation of the fibrous structure. As shown in Figure 1, zein not only formed nanoparticles, but also just formed a crinkled shape at low concentration. On the contrary, when we used zein with a 8/2 ethanol/H_2_O ratio (Figure 1), nanoparticles were easily obtained at 10 wt % zein concentration. Another concentration of zein showed a more beaded morphology than zein in because of the low viscosity of the spraying solution. The reason for obtaining different shapes at each concentration is that the Raleigh forces, which assist in particle formation, were able to overcome the viscous forces to enable the formation of particles [42]. At high zein concentration (above 20 wt %), the zein nanomaterials exhibited a fiber shape. The FE-SEM results indicate that uniform nanoparticles were obtained from the 8/2 (*v/v*) ethanol/water solvent with a zein concentration of 10 wt %. FE-SEM and TEM images of the prepared zein/Ag composite nanoparticles with various Ag content are presented in Figure 2 and Figure 3, respectively. Our previous work indicated that the 10 wt % zein solution in an 8/2 (*v/v*) ethanol/water mixture solvent was the optimum polymer concentration for producing uniform zein nanoparticles by electrospinning [41]. Figure 2 presents the FE-SEM images obtained from electrospun zein nanoparticles containing 0, 2 and 4 wt % Ag nanoparticles. Figure 2 demonstrates that the addition of even a very small amount of Ag nanoparticles affects the morphology of the zein/Ag composite nanoparticles, causing the zein nanoparticles to crinkle up and, consequently, contract, which might be caused by the increase in the solution concentration and viscosity (Table 2). This shrinkage of the zein/Ag nanoparticles increased slightly with increasing Ag content. Moreover, a few nanofibers connected with nanoparticles are observed in Figure 2c, which were obtained from the 10 wt % zein solution containing 4 wt % Ag nanoparticles. The additional viscosity and increased concentration with increasing Ag content might cause these morphological changes.

Figure 3 presents the TEM images of single isolated zein/Ag composite nanoparticles containing 0, 2 and 4 wt % Ag nanoparticles. These TEM images indicate the coexistence of Ag nanoparticles and a zein matrix in the electrospun zein/Ag composite nanoparticles. Moreover, these images indicate that the nanoparticles contracted and were reshaped with increasing Ag content. Due to the strong interactions between the polymer and metal, the Ag nanoparticles are embedded well within the zein matrix. The TEM images indicate that the Ag nanoparticles are well dispersed in the polymer matrix and that the number of Ag nanoparticles gradually increases in the zein/Ag composite nanoparticles with increasing Ag content. These results support the FE-SEM results described above. It is evident from the FE-SEM and TEM results that a small amount of Ag nanoparticles can effectively alter the morphology of zein nanoparticles.

### 3.2. XRD Data

**Figure 4:**
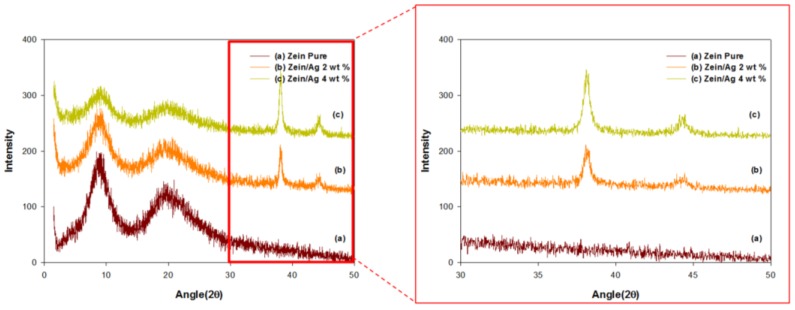
X-ray diffraction patterns of zein/Ag nanoparticles electrospun from ethanol aqueous solutions with an ethanol/water ratio of 8/2 (*v/v*) at Ag concentrations of (**a**) 0, (**b**) 2 and (**c**) 4 wt % (polymer concentration = 10 wt %, TCD = 15 cm, and applied voltage = 15 kV).

XRD patterns of the electrospun nanoparticles obtained from pristine zein and the zein/Ag blend systems with various Ag concentrations are presented in Figure 4. The pure zein powder exhibits a sharp peak at 2θ = 8.96° (9.86 Å) and a broad peak at approximately 2θ = 19.58° (4.53 Å) [43]. The shorter d-spacing of approximately 4.5 Å is believed to be related to the average backbone distance within the α-helix structure of zein, whereas the larger d-spacing of approximately 10 Å is thought to be the spacing of the inter-helix packing or the mean distance of approach of neighboring helices, as described in the literature [44]. The electrospun zein nanoparticles in this work exhibited diffraction peaks that were similar to those of the pure zein powder (Figure 4a). This result indicates that the zein retains its structural conformation in the electrospun nanoparticles. The XRD patterns of the zein/Ag composite nanoparticles show diffraction peaks near 8.96°, 19.58°, 38.2° and 44.6°. However, only the zein nanoparticles do not exhibit any diffraction peaks at 2θ values higher than 19.58° (Figure 4a). Hence, except for the diffraction peaks of zein (2θ = 8.96° and 19.58°), all of the other peaks originated from the Ag phase (Figure 4b,c). These peaks correspond to the (111) and (200) planes of Ag nanocrystals with cubic symmetry [45]. The diffraction peaks of the zein/Ag composite nanoparticles near 2θ values of 8.96° and 19.58° (Figure 4b,c) indicate that the structural conformation of zein remains unaltered in the composite nanoparticles with Ag. This structural stability of zein indicates the coexistence of Ag nanoparticles with the zein matrix, which was also supported by the TEM results. Moreover, the XRD patterns clearly demonstrate that the diffraction peak intensities corresponding to Ag nanoparticles increased and those related to zein decreased and broadened with increasing Ag content in the zein/Ag composite nanoparticles. These changes in peak intensity also suggest the significant congregation of Ag nanoparticles with increasing Ag content within the zein/Ag composite nanoparticles. This was also supported by the TEM results.

### 3.3. Thermal Stability

**Figure 5:**
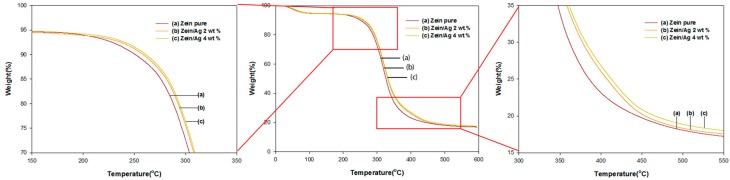
Thermogravimetric analysis data of zein/Ag nanoparticles electrospun from ethanol aqueous solutions with an ethanol/water ratio of 8/2 (*v/v*) at Ag concentrations of (**a**) 0, (**b**) 2 and (**c**) 4 wt % (polymer concentration = 10 wt %, TCD = 15 cm, and applied voltage = 15 kV).

Electrospun zein nanomaterials are more thermally stable than solution cast films [10]. Moreover, the introduction of inorganic materials into the electrospun zein nanocomposites also improves their thermal stability [11]. The thermal stability of the electrospun zein/Ag nanoparticles was examined using TGA in a nitrogen atmosphere to investigate the effect of the Ag nanoparticles on the thermal stability of the composites. Figure 5 presents the TGA thermograms obtained from zein/Ag nanoparticles containing 0, 2 and 4 wt % Ag nanoparticles at various decomposition temperatures. Three typical weight losses were observed in the TGA thermograms of both pure zein and the zein/Ag nanocomposites [10,11]. The top plot (Figure 5a) represents the pristine zein nanoparticles and the bottom plot (Figure 5c) represents the zein/Ag composite nanoparticles containing 4 wt % Ag nanoparticles, i.e., the highest mass ratio of Ag used in this work. Figure 5b displays the TGA curve for the 2 wt % Ag content, which shows the same trend in thermal stability as that shown in Figure 5a,c. The TGA thermograms indicate that the addition of Ag nanoparticles to the electrospun zein nanoparticles significantly increased the thermal stability of the zein nanoparticles, and that this thermal stability increased considerably depending on the Ag content. Notably, the thermal stability increase corresponds to the amount of Ag nanoparticles throughout the thermal decomposition process. The additional thermal stability of the electrospun zein/Ag composite nanoparticles at comparatively high Ag content might be attributed to their higher chain compactness due to the interaction between the zein and the Ag nanoparticles. This result clearly indicates that Ag nanoparticles have a positive effect on the thermal stability of the electrospun zein nanoparticles.

### 3.4. Antibacterial Efficacy

**Figure 6:**
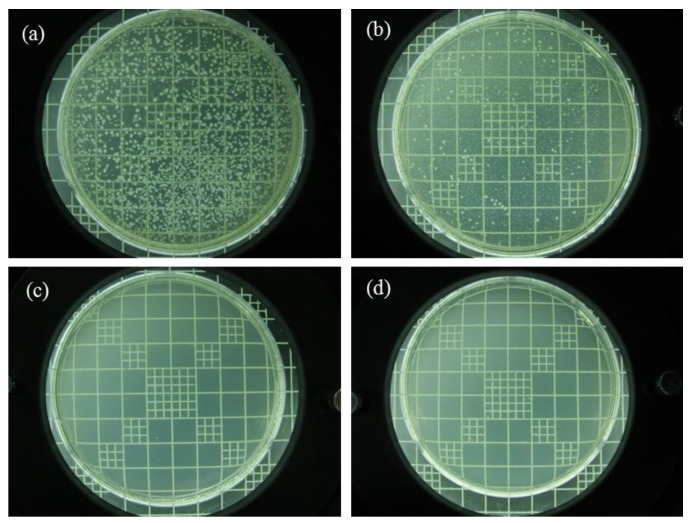
Antibacterial ability against *Staphylococcus aureus*. (**a**) blank and with zein/Ag nanoparticles containing Ag concentrations of (**b**) 0 wt %; (**c**) 2 wt %; and (**d**) 4 wt % (after one week).

**Figure 7:**
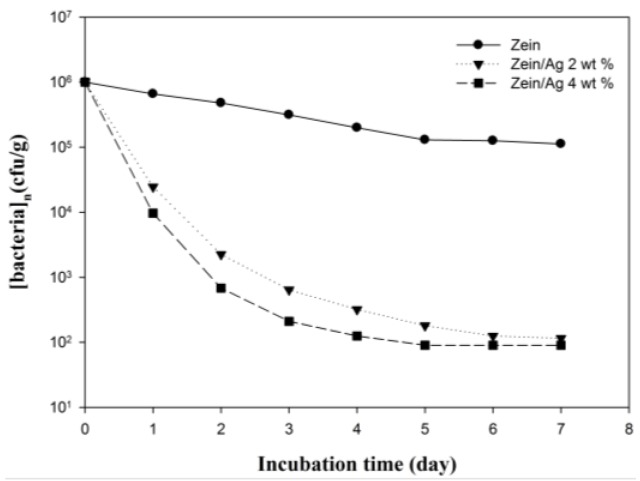
Preservation performance of zein/Ag nanoparticles prepared with different Ag concentrations of 0, 2 and 4 wt %.

**Figure 8:**
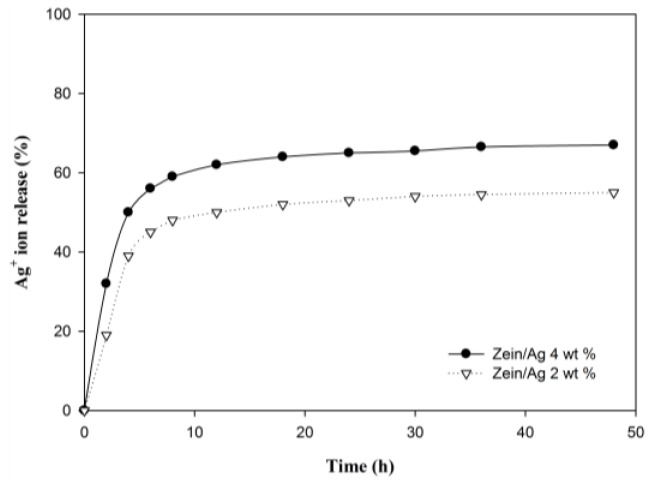
Ag^+^ release from zein/Ag nanopartilces for different periods of time. The concentration of incorporated Ag nanoparticle in zein were 2 wt % and 4 wt %.

Ag nanoparticles are known to exhibit strong inhibitory and antibacterial effects, as well as a broad spectrum of antimicrobial activities [5,46]. To provide useful information on the biological function of electrospun zein/Ag composite nanoparticles, the antibacterial performance of the electrospun zein and zein/Ag composite nanoparticles was evaluated in viscous aqueous test samples. The results obtained against *Staphylococcus aureus* are presented in Figure 6. The antibacterial efficacy of the pure zein and zein/Ag composite nanoparticles was assessed by counting the number of bacteria remaining in the sample after a certain time of storage at 32 °C. As observed in Figure 6b, the pure zein nanoparticles exhibited antibacterial properties; however, they could not fully prevent bacterial growth. This is because the antibacterial effect of the zein polymer, which is resistant to microbial attack [9]. In contrast, the incorporation of the Ag composite nanoparticles into the test samples resulted in a remarkable decrease in the number of bacteria, as observed in Figure 6c,d. Based on the Ag ion release, it is expected that all the zein/Ag nanoparticles investigated exhibit good antimicrobial efficacy. Sterilized specimens of the zein, as well as of the Ag-filled samples, were inoculated with a suspension of *Staphylococcus aureus* in Luria broth to check their antimicrobial efficacy. The Ag content in the zein/Ag composite nanoparticles shows no significant trend in reducing the number of bacteria. With only a small amount of Ag, almost all the initially inoculated bacteria could be sterilized within a week. This result indicates that the electrospun zein/Ag nanoparticles are highly effective antibacterial materials and suggests that only a small amount of Ag nanoparticles can make zein more efficient against bacteria. In order to provide useful information on the biological function of zein and zein/Ag nanoparticles, the antibacterial performance of zein and zein/Ag was evaluated in viscous aqueous test samples, and the results are shown in Figure 7. As shown in Figure 7, the zein without Ag show little antibacterial performance (bacteriostatic ratio of zein = 80%). The increase in the concentration of the Ag nanoparticles accelerate diminishing in bacterial (bacteriostatic ratio of zein/Ag = 99.99%). The number of bacteria in the test samples remained constant for a long time. In contrast, the incorporation of Ag nanoparticles showed a remarkable decrease in the number of bacteria. Increasing the Ag concentration in the zein/Ag composite nanoparticles enhanced the decline in the level of bacteria. With only a small amount of Ag present, almost all of the initially inoculated bacteria could be sterilized within a week. This indicates that the zein/Ag nanoparticles have an antibacterial activity. The release of Ag from zein/Ag nanoaprticles during different periods of time is shown in Figure 8. A similar behavior is observed for both the nanoparticles, although the Ag^+^ ion is higher for zein/Ag 4 wt %. After 10 h of incubation, the ratio of Ag^+^ ion release nearly stabilized. The result of antibacterial activity and Ag^+^ ion release indicates that Ag^+^ ion attacks the bacterial activity at the early time.

## 4. Conclusions

Zein/Ag composite nanoparticles were successfully electrospun from zein solutions containing variable amounts of Ag nanoparticles to investigate the effect of Ag nanoparticles on the properties and morphologies of zein nanoparticles. The addition of only a small amount of Ag nanoparticles was effective enough to change the structures and properties of the zein nanoparticles. The addition of Ag nanoparticles caused the zein nanoparticles to slightly contract and crinkle. The Ag nanoparticles were observed to co-exist within the zein matrix without changing the helical conformation of zein. The addition of Ag nanoparticles significantly enhanced the thermal stability of the zein nanoparticles. The zein/Ag composite nanoparticles performed better than the pure zein nanoparticles against bacterial growth. These results suggest the potential practical use of zein/Ag composite nanoparticles.

## Figures and Tables

**Table 1:** The viscosity value of electrospun zein nanomaterials from ethanol aqueous solutions with ethanol/water ratios of 7/3, 8/2 and 9/1 (*v/v*) according to polymer concentrations of 10, 15 and 20 wt %, respectively (Tip-to-collector distance (TCD) = 15 cm and applied voltage = 15 kV) at 25 °C.

Concentration	10 wt %	15 wt %	20 wt %
**EtOH/H_2_O = 7/3**	8.7 ± 1.6 mPa·s	20.8 ± 2.6 mPa·s	38.2 ± 2.7 mPa·s
**EtOH/H_2_O = 8/2**	9.1 ± 1.8 mPa·s	28.7 ± 2.1 mPa·s	45.2 ± 3.4 mPa·s
**EtOH/H_2_O = 9/1**	9.8 ± 1.4 mPa·s	35.1 ± 2.7 mPa·s	52.1 ± 3.5 mPa·s

**Table 2:** The viscosity of zein/Ag nanoparticles electrospun from ethanol aqueous solutions with an ethanol/water ratio of 8/2 (*v/v*) according to Ag concentrations of 0, 2 and 4 wt % (polymer concentration = 10 wt %, TCD = 15 cm, and applied voltage = 15 kV) at 25 °C.

Ag Concentration	0 wt %	2 wt %	4 wt %
**EtOH/H_2_O = 8/2**	9.1 ± 1.8 mPa·s	11.8 ± 2.1 mPa·s	12.4 ± 2.6 mPa·s
